# Diacerein reduces inflammasome activation and SARS-CoV-2 virus replication: a proof-of-concept translational study

**DOI:** 10.3389/fphar.2024.1402032

**Published:** 2024-10-07

**Authors:** Helison R. P. Carmo, Alejandro Rossel Castillo, Isabella Bonilha, Erica I. L. Gomes, Joaquim Barreto, Filipe A. Moura, Gustavo Gastão Davanzo, Lauar de Brito Monteiro, Stéfanie Primon Muraro, Gabriela Fabiano de Souza, Joseane Morari, Flávia Elisa Galdino, Natália S. Brunetti, Guilherme Reis-de-Oliveira, Victor Corasolla Carregari, Wilson Nadruz, Daniel Martins-de-Souza, Alessandro S. Farias, Licio A. Velloso, José Luiz Proenca-Modena, Marcelo A. Mori, Watson Loh, Deepak L. Bhatt, Derek M. Yellon, Sean M. Davidson, Pedro G. De Oliveira, Pedro M. Moraes-Vieira, Andrei C. Sposito

**Affiliations:** ^1^ Laboratory of Vascular Biology and Atherosclerosis (Aterolab), State University of Campinas (UNICAMP), Campinas, Brazil; ^2^ Brigham and Women’s Hospital, Division of Cardiovascular Medicine, Brigham and Women’s Hospital, Harvard Medical School, Boston, MA, United States; ^3^ TIMI Study Group, Division of Cardiovascular Medicine, Brigham and Women’s Hospital, Harvard Medical School, Boston, MA, United States; ^4^ Laboratory of Immunometabolism, Department of Genetics, Evolution, Microbiology and Immunology, Institute of Biology, University of Campinas (UNICAMP), Campinas, Brazil; ^5^ Laboratory of Emerging Viruses, Department of Genetics, Microbiology and Immunology, Institute of Biology, University of Campinas (UNICAMP), Campinas, Brazil; ^6^ Laboratory of Cell Signaling, Obesity and Comorbidities Research Center, University of Campinas (UNICAMP), Campinas, Brazil; ^7^ Institute of Chemistry, University of Campinas (UNICAMP), Campinas, Brazil; ^8^ Brazilian Synchrotron Light Laboratory (LNLS), Brazilian Center for Research in Energy and Materials (CNPEM), Campinas, Brazil; ^9^ Autoimmune Research Laboratory, Department of Genetics, Microbiology and Immunology, Institute of Biology, University of Campinas (UNICAMP), Campinas, Brazil; ^10^ Laboratory of Neuroproteomics, Institute of Biology, University of Campinas (UNICAMP), Campinas, Brazil; ^11^ D’Or Institute for Research and Education (IDOR), São Paulo, Brazil; ^12^ Experimental Medicine Research Cluster (EMRC), University of Campinas (UNICAMP), Campinas, Brazil; ^13^ Laboratory of Aging Biology, Department of Biochemistry and Tissue Biology, Institute of Biology, University of Campinas, Campinas, Brazil; ^14^ Mount Sinai Fuster Heart Hospital, Icahn School of Medicine at Mount Sinai, New York, NY, United States; ^15^ The Hatter Cardiovascular Institute, University College London, London, United Kingdom; ^16^ Instituto de Ortopedia e Traumatologia, Hospital das Clinicas HCFMUSP, Faculdade de Medicina, Universidade de São Paulo (USP), São Paulo, Brazil; ^17^ Sport Traumatology Group, Department of Orthopaedics and Traumatology, Santa Casa de São Paulo School of Medical Sciences, São Paulo, Brazil

**Keywords:** COVID-19, diacerein, rhein, pre-clinical, clinical trial

## Abstract

**Background:**

Severe acute respiratory syndrome coronavirus 2 (SARS-CoV-2) is linked to high mortality, primarily through an intense inflammatory response. Diacerein has emerged as a potential therapy for COVID-19 due to its potential impact in decreasing the inflammasome activation and coronavirus replication. This study aims to explore diacerein’s influence in inhibiting both viral replication and the inflammatory response after SARS-CoV-2 infection.

**Methods:**

Human peripheral blood mononuclear cells (PBMCs) were obtained from healthy volunteers and infected *in vitro* with SARS-CoV-2. Additionally, we carried out a pilot randomized, double-blind, placebo-controlled study with 14 participants allocated to diacerein (n = 7) or placebo (n = 7) therapies every 12 h for 10 days. The primary endpoint was change in plasma markers of inflammasome activation (NLRP3, caspase-1, and gasdermin-D).

**Results:**

*In vitro* protocols have shown that rhein, diacerein’s primary metabolite, decreased IL-1β secretion caused by SARS-CoV-2 infection in human PBMCs (*p* < 0.05), and suppressed viral replication when administered either before or after the virus incubation (*p* < 0.05). This later effect was, at least partially, attributed to its inhibitory effect on 3-chymotrypsin-like protease (SARS-CoV-2 3CL^pro^) and papain-like protease in the SARS-CoV-2 (SARS-CoV-2 PL^pro^) virus and in the phosphorylation of proteins related cytoskeleton network (*p* < 0.05). Diacerein-treated COVID-19 patients presented a smaller area under the curve for NLRP3, caspase-1 and GSDM-D measured on days 2, 5, and 10 after hospitalization compared to those receiving a placebo (*p* < 0.05).

**Conclusion:**

The indicated mechanisms of action of diacerein/rhein can reduce viral replication and mitigate the inflammatory response related to SARS-CoV-2. These findings are preliminary and require confirmation in clinical trials.

## 1 Introduction

Diacerein [4,5-Bis(acetyloxy)-9,10-dihydro-9,10-dioxo-2-anthracenecarboxylic acid], also known as diacetylrhein, is a prodrug of the anthraquinone class obtained from Aloe Vera plants (*Aloe barbadensis* Miller) used to treat osteoarthritis. Rhein, the active metabolite of diacerein, reduces the cellular inflammatory response through a wide range of mechanisms, including membrane desensitization of interleukin-1 (IL-1) receptors, inhibition of the transcriptional regulatory pathway mitogen-activated protein kinase (MEK)/extracellular signal-regulated kinases (ERK), and inhibition of IL-1β activation through modulation of caspase-1 (also named IL-1 converting enzyme, ICE) enzymatic activity (Henamayee et al., 2020). These multiple anti-inflammatory actions led to the recent proposal that it may be potentially beneficial to patients with coronavirus disease 2019 (COVID-19) (de Oliveira et al., 2020). In this context, pre-clinical studies with human peripheral blood mononuclear cells (PBMCs) have helped to discover new therapeutic avenues to attenuate the pro-inflammatory response resulting from severe acute respiratory syndrome coronavirus 2 (SARS-CoV-2) infection. PBMCs can respond to a cytokine stimulus similar to that induced by SARS-CoV-2 infection in a systemic inflammation scenario (Kazmierski et al., 2022). For this reason, this *in vitro* model has been utilized in immunological and transcriptomics studies (Manunta et al., 2021).

Studies using cell models suggest that the activation of the NLRP3 inflammasome is one of the pathways contributing to the rise in systemic inflammation SARS-CoV-2 infection (Rodrigues et al., 2021). This protein complex is composed of nucleotide-binding oligomerization domain, leucine-rich repeat, and pyrin domain-containing 3 (NLRP3) associated with adapter apoptosis-associated speck-like protein containing a CARD (ASC) and pro-caspase-1 (Rodrigues et al., 2021). The NLRP3 inflammasome activation leads to lytic cell death (also named pyroptosis), executed via the cleavage of the gasdermin-D membrane protein (GSDM-D), as well as the activation/secretion of pro-IL1β and pro-IL18 (Rodrigues et al., 2021; Vora et al., 2021; Zhou et al., 2022). Furthermore, fragments of cells or the presence of viral proteins (e.g., ORF3a, ORF-3b, nucleocapsid, N protein, E envelope protein) can be recognized as damage-associated molecular patterns (DAMPs) by NLRP3 inflamassome within adjacent cells, thus propagating the extension of the lesion (Vora et al., 2021).

In this study, diacerein/rhein was tested in preclinical protocols *in vitro* as well as in a pilot randomized double-blind trial with COVID-19 patients. The aim was to assess the biological plausibility of the effect of diacerein/rhein on the inflammatory response associated with the NLRP3 inflammasome pathway and on viral replication.

## 2 Materials and methods

### 2.1 Study population

Patients with confirmed mild to moderate COVID-19 according to the World Health Organization’s Clinical Progression Scale (WHO-CPS) were recruited from Unicamp Clinical Hospital in Campinas, Brazil (WHO, 2020). Given the absence of previous studies on diacerein or specific caspase-1 inhibitors in the context of COVID-19, this study was conceived as a pilot test to establish its conceptual feasibility, hence, there was no calculation of sample size. The study initially planned for 40 participants, was halted at 14 participants due to a substantial decrease in Covid-19-related hospitalizations. The clinical trial has received ethical approval from the Ethics Committee of the State University of Campinas (CAAE: 50440921.6.0000.5404) and was registered at ClinicalTrials.gov (NCT05226754).

### 2.2 Randomization and blinding

Following enrollment, patients were randomly assigned in a 1:1 ratio to receive either diacerein 50 mg or a placebo treatment every 12 h for 10 days, regardless of their hospitalization status. The research electronic data capture (REDCap^®^) platform served as our randomization system. To maintain the integrity of the study, patients, investigators, and support staff were kept blinded to the experimental therapy. Diacerein 50 mg and placebo capsules, identical in appearance to ensure effective blinding, were supplied by TRB Pharma, SP, Brazil. All laboratory analyses were conducted without knowledge of the treatment assignment. Identification of the study drug only took place upon unlocking the dataset, preserving the blinding throughout the study.

### 2.3 Clinical trial endpoints

Our primary objective was to investigate whether diacerein therapy could mitigate the elevation of plasma markers linked to NLRP3 inflammasome activation. We defined as endpoints the area-under-the-curve (AUC) for the measurements of IL-1β, nucleotide-binding oligomerization domain, leucine-rich repeat, and pyrin domain-containing 3 (NLRP3), gasdermin-D (GSDM-D), and caspase-1 concentrations changes from the baseline.

### 2.4 Human peripheral blood mononuclear cells protocol isolation for *in vitro* experiments

The *in vitro* stage of the study was carried out with the approval of the Brazilian Committee for Ethics in Human Studies (CAEE: 31622420.0.0000.5404). Peripheral blood mononuclear cells (PBMCs) from healthy donors were tested negative for COVID-19, provided by the Hematology and Hemotherapy Center of the University of Campinas, Brazil. In short, blood samples were diluted in phosphate-buffered saline (PBS) at a ratio of 1:1 in a ficoll-hypaque density gradient and subjected to centrifugation (2,700 rpm) for 20 min at room temperature to isolate and obtain mononuclear cells. Approximately 1.5 × 10^6^/mL were seeded in RPMI-1640 culture medium in a cell culture plate for 2 h. Followed cells were washed with PBS to avoid non-adherent ones and incubated with 10% bovine serum at 37°C with 5% CO_2_ atmosphere.

### 2.5 Pharmacologic approaches

Rhein or 4,5-dihydroxy-9,10-dioxoanthracene-2-carboxylic acid (R7269, Sigma-Aldrich); VRT-043198 (244133-31-1, MedKoo Biosciences Inc.); and Emricasan (254750-02-2, MedKoo Biosciences Inc.) were used*in vitro* protocols with human PBMCs and protein activity assays.

### 2.6 Viability assay

The cells were seeded in a 24-well culture plate and cultivated until the experimental protocol in a cell culture incubator at 37°C. The viability protocol was performed using a flow cytometry system according to the Viability Stain 510 assay kit datasheet (BD Horizon Fixable^®^).

### 2.7 Infection protocol

A strain of the virus isolated and cultivated by researchers at the Institute of Biomedical Sciences of the University of São Paulo (ICB-USP) from patients with COVID-19 in Brazil, characterized by RNA sequencing method: HIAE-02 virus was used. SARS-CoV-2/SP02/human/2020/BRA (GenBank: MT126808.1). In a biosafety level 3 (NB3) laboratory, cultured mononuclear cells were infected for 60 min at a multiplicity of infection (MOI) 0.1 with SARS-CoV-2 or mock (control) dispersed in RPMI-1640 enriched culture medium and maintained in constant agitation. Protocol validated at the Laboratory of Studies of Emerging Viruses of the Department of Genetics, Evolution, Microbiology, and Immunology of the Institute of Biology at UNICAMP (Codo et al., 2020).

### 2.8 RNA extraction protocol

RNA extraction used a guanine solution (Trizol™) with 5-min incubation. RNAse-free chloroform (200 µL per 1 mL Trizol) was added, agitated, and then centrifuged (13,000G, 15 min, 4°C). After phase separation, the supernatant was collected, isopropanol added (10 min, room temperature), and centrifuged (13,000G, 10 min, 4°C). Supernatant mixed with ethanol (70%) was again centrifuged (13,000G, 5 min, 4°C), and the pellet was resuspended in 12 µL RNAse-free water. 1µL of this material was used for spectrophotometry (260–280 nm) to assess RNA purity and concentration. Reverse transcription used 1 μg total RNA with a GoScript Reverse kit, following the manufacturer’s protocol.

### 2.9 Viral load detection and human gene expression

The specific SARS-CoV-2 N1 primer targeting the N1 region was used (Codo et al., 2020). The standard curve was performed by serial dilutions of SARS-CoV-2. Viral load and human gene expression were quantified by PCR-RT (BIO-RAD CFX394) in 384-well plates with hydrolysis probes (SybrGreen Supermix). All primers except SARS-CoV-2 N1 were obtained from Integrated DNA Technologies, Inc. (Coralville, Iowa, EUA) (Table 1).

**TABLE 1:** The primers sequences were for human gene expression. Nucleotide-binding oligomerization domain, leucine-rich repeat, and pyrin domain-containing 3 (NLRP3); gasdermin-D (GSDMD); apoptosis-associated speck-like protein containing a CARD (ASC); caspase-1 (CASP1); interleukin 1β (IL1β); and interleukin 18 (IL18).

NLRP3	Forward 5′-GAA​TGC​CTT​GGG​AGA​CTC​AG-3′
	Reverse 5′-AGA​TTC​TGA​TTA​GTG​CTG​AGT​ACC-3′
GSDMD	Forward 5′-GAA​CTG​AGT​GTG​GAC​AGA​GC-3′
	Reverse 5′-CTGAGGCCAGTATGCTGAAG-3′
CASP1	Forward 5′-AGA​TCA​AAC​ATC​TGG​AAA​TTA​CCT​T-3′
	Reverse 5′-CAA​AGC​TTG​ACA​TTC​CCT​TCT​G-3′
ASC	Forward 5′-CTC​ACC​GCT​AAC​GTG​CTG-3′
	Reverse 5′-CAC​TCA​ACG​TTT​GTG​ACC​CT-3′
IL1β	Forward 5′-CAGCCAATCTTCATTGCTCAAG-3′
	Reverse 5′-GAA​CAA​GTC​ATC​CTC​ATT​GCC-3′
IL18	Forward 5′-CAG​ACC​TTC​CAG​ATC​GCT​TC-3′
	Reverse 5′-AAT​TTC​ATT​GCC​ACA​AAG​TTG​ATG-3′

### 2.10 Protocol for priming and activating of human peripheral blood mononuclear cells

Human PBMCs were initially counted and seeded into a 48-well culture plate. Subsequently, they were incubated in RPMI 1640 medium supplemented with 10% fetal bovine serum (FBS), 1% glutamine, and 1% penicillin-streptomycin at 37°C in a 5% CO_2_ atmosphere. The human PBMCs underwent a sequential drug exposition: firstly, 3 h of pretreatment with lipopolysaccharides (100 ng/mL; Sigma-Aldrich) followed by a 1-h exposure to nigericin (10µM; Sigma-Aldrich) at 37°C in a 5% CO_2_ atmosphere. During the drug exposition, the human PBMCs were divided into four distinct groups: (I) control (medium only), (II) rhein (60 µM) during lipopolysaccharide (LPS) stimulation, (III) rhein (60 µM) during nigericin stimulation, and (IV) VRT-043198 (0.5 µM) during nigericin stimulation. Human PBMCs underwent a proinflammatory response induced by two complementary signals. Firstly, the signal-1 involved a 3-h stimulation with LPS to upregulate NLRP3, ASC, caspase-1 and IL-1β genes, generating NLRP3 post-translational licensing and inflammasome assembly. Secondly, the signal-2 involved 1-h stimulation with nigericin, acting as an inducer for NLRP3 inflammasome activation. The human PBMCs were maintained with their respective treatments for 24 h. Upon completion of the treatment period, the plate was centrifuged at 1,200 rpm for 5 min, and the supernatant was collected and stored at −80°C until the IL-1β dosage. The human PBMCs were harvested and prepared for flow cytometry assay using a viability kit (Zombie Aqua™ Fixable, Biolegend) and the proliferation intracellular marker Ki-67 (eFluor 660, Invitrogen). Briefly, the cells were incubated with 40uL per sample of the viability marker (diluted at 1:1,000) in PBS for 30 min in a light-protected environment at room temperature. Subsequently, the samples underwent a single wash with 1 mL of PBS supplemented with 2% FBS and were centrifuged at 1,200 rpm for 10 min at 4°C. The supernatant was discarded, and the cells were fixed and permeabilized using 200uL of Fixation/Permeabilization solution (BD Cytofix/Cytoperm™) for 20 min at 4°C. Following, another wash with 1 mL of wash buffer (BD Perm/Wash™) was performed using the same centrifugation protocol mentioned earlier. The cell pellets were then resuspended in 100uL of a solution containing the Ki-67 intracellular antibody (1:100 dilution) and incubated at 4°C overnight. The day after, the samples were washed with wash buffer and resuspended in 300uL of PBS. All flow cytometry analyses were conducted using a fluorescence-activated cell sorting (FACS) analyzer (Symphony A5, BD Biosciences), and the data were analyzed using FlowJo software (v10).

### 2.11 Enzyme-linked immunosorbent assays (ELISA) and protease activity measurements

According to the manufacturer’s recommendations, the following ELISA measurements were performed with IL1-β with the kit package insert human IL-1β/IL-1F2 (Duo Set ELISA) from R&D system; human NLRP3 (Nod Like Receptor Pyrins-3, EH4202) and human GSDMD (gasdermin-D, EH8956) with the kits from Fine-Test; and Human caspase-1 from ABclonal (RK01035). The activity of the 3CL protease, MBP-tag (SARS-CoV-2; catalogue 79955-1) and papain-like protease (SARS-CoV-2; catalogue 79995) were both from BPS bioscience. Finally, a caspase-1 drug discovery kit (BML-AK701) was used to assess its direct inhibition by rhein and VRT-043198.

### 2.12 Isothermal titration calorimetry

Calorimetric experiments were performed using a MicroCal PEAQ-ITC (Malvern). All solutions were prepared in PBS. SARS-CoV-2 S1 protein (2019-nCoV S1 protein, GenScript, Cat. 434 No. Z03501) and rhein (R7269, Sigma-Aldrich) were used. The injection syringe and sample cell were filled with 40 µL of 5 µM solution of rhein and 200 µL of 300 nM solution of S1, respectively. Aliquots of 2 or 3 µL of rhein solution were injected in the sample cell every 300 s and under stirring of 750 rpm and at 37°C. The dilution experiment was then conducted by titrating rhein into PBS under the same conditions to discount its heat effect to determine the heat of interaction per injection. The incremental heat was obtained after adjusting the baseline and integrating the heat using the MicroCal PEAQ-ITC analysis software.

### 2.13 Proteomic analysis

Human PBMCs infected with SARS-CoV-2, rhein-exposed, and control cells were suspended in radioimmunoprecipitation assay (RIPA) buffer containing 150 mM NaCl, 1 mM EDTA, 100 mM Tris-HCL, and 1% Triton-X as well as protease and phosphatase inhibitors (Protease Inhibitor Cocktail, Sigma-Aldrich). After subjecting them to three cycles of 30 s of ultrasonication for mechanical cell lysis, the protein content was quantified using the Pierce BCA protein assay kit (Thermo Scientific). To improve protein yield, we employed the filter-aided sample preparation (FASP) protocol for subsequent analyses (Distler et al., 2016). This protocol concentrates proteins and purifies samples through a series of washing steps in a microcolumn tip with a 10 kDa MW cutoff. Tryptic digestion is also performed in this column. We used 10 µg of protein for the FASP protocol, which includes reduction, alkylation, and trypsin digestion steps.

### 2.14 Phospho-enrichment

The mixed peptide sample was diluted tenfold with a loading buffer comprising 80% acetonitrile, 5% trifluoroacetic acid, and 1M glycolic acid. In this solution, 5 mg of TiO_2_ beads (1 mg TiO_2_ per 100 μg of peptide) was added and agitated for 10 min at 600 rpm, followed by centrifugation. The supernatant was incubated with half the amount of TiO_2_ and then was lyophilized. The TiO_2_ beads were subjected to a series of washes: (i) 100 μL of loading buffer (15-s mixing), (ii) 100 μL of washing buffer A (comprising 80% Acetonitrile and 1% Trifluoroacetic acid), and (iii) 100 μL of washing buffer B (consisting of 20% acetonitrile and 0.2% trifluoroacetic acid). Phosphopeptides were eluted from the TiO_2_ resin using 50 μL of eluting buffer (40 μL of 28% ammonia solution in 980 μL of water, pH 11.3) and centrifuged for 1 min. The eluted phosphopeptides were collected and passed through a C8 stage tip to remove TiO_2_ beads, and the phosphopeptides attached to the C8 tip were subsequently eluted with 1 μL of 30% Trifluoroacetic acid. Finally, the phosphopeptides were lyophilized (Dominguez et al., 2021).

### 2.15 Liquid chromatography-tandem mass spectrometry analysis

Each sample, consisting of 1 µg of digested material, was loaded onto a Symmetry C18 5 μm, 180 μm × 20 mm precolumn (Waters Corp. Milford, MA, United States). The samples underwent separation through a 120-min reversed-phase gradient at 300 nL/min, characterized by a linear gradient from 3% to 80% CH_3_CN over 90 min, using a HSS T3 C18 1.8 μm, 75 μm × 150 mm nanoscale LC column (Waters Corp. Milford, MA, United States) maintained at 40°C. The ionized peptides were then acquired using a Synapt G2-Si mass spectrometer (Waters Corp. Milford, MA, United States).

Shotgun proteomic analysis was conducted via data-independent acquisition (DIA), employing “expression” configuration mode with the ion mobility cell (HDMSe). In ion mobility mode, a wave velocity of 1000 m/s for ion separation and a transfer wave velocity of 175 m/s were applied. The mass spectrometer operated in “expression mode” alternating between low (4 eV) and high (25–60 eV) collision energies on the gas cell, with a scan time of 1 s per function across the 50–2000 m/z range. The processing encompassed both low and high energy, coupled with data from the reference lock mass ([Glu1]-Fibrinopeptide B Standard, Waters Corp. Milford, MA, United States), yielding a time-aligned inventory of accurate mass-retention time components for both low and high energy, exact mass retention time (EMRT).

The samples were analyzed in three technical replicates. Continuum liquid chromatography-mass spectrometry (LC-MS) data from three replicate experiments for each sample were processed using Progenesis QC for proteomics software (PLGS, Waters Corp. Milford, MA, United States) for protein identification and quantification. Proteins were identified by searching the *Homo sapiens* database (UniProt KB/Swiss-Prot Protein reviewed 2022) with the following parameters: automatic tolerance for precursor and product ions, a minimum of 1 fragment ion match per peptide, a minimum of 3 fragment ion matches per protein, a minimum of 1 unique peptide match per protein, 2 missed cleavages, carbamidomethylation of cysteines as a fixed modification, oxidation of methionines, and phosphorylation of STY residue as variable modifications, with a false discovery rate (FDR) of the identification algorithm set to <1%. Label-free quantitative analysis was performed using relative abundance intensity in Progenesis software, incorporating all identified peptides for normalization. The expression analysis considered technical replicates for each experimental condition, treating each group as an independent variable.

### 2.16 Statistical analysis

The assessment of efficacy involved a comparison of study arms, as per the intention-to-treat analysis, focusing on the AUC for the values on days 0, 2, 5 and 10, adjusted for the admission value to mitigate the regression to the mean effect. These analyses were performed using the rank covariance analysis (RANKOVA), for each of the inflammasome markers measured in the trial. We refrained from conducting analyses at each collection point to preserve statistical power, recognizing that assessing the response as the total exposure during hospitalization is better estimated using AUC. In our *in vitro* experiments, statistical evaluations involving two groups were conducted using the *t*-test, while analyses involving three or more groups were performed using the one-way ANOVA followed by Tukey’s *post hoc* test. The Kruskal–Wallis test was used to assess the 3-chymotrypsin-like and papain-like proteases of SARS-CoV-2, followed by Dunn’s test for multiple comparisons. Shotgun proteomic analysis was generated to include proteins found in at least two out of three technical replicates while excluding proteins with less than a 20% change and those lacking statistical significance (ANOVA) with the Tukey *post hoc* test. All analyses were carried out using SPSS version 25 and R version 3.5.2 for Mac. A *p*-value of less than 0.05 was considered indicative of statistical significance.

## 3 Results

### 3.1 Rhein reduces the gene expression and secretion of IL-1beta in human PBMCs infected with SARS-CoV-2

**FIGURE 1:**
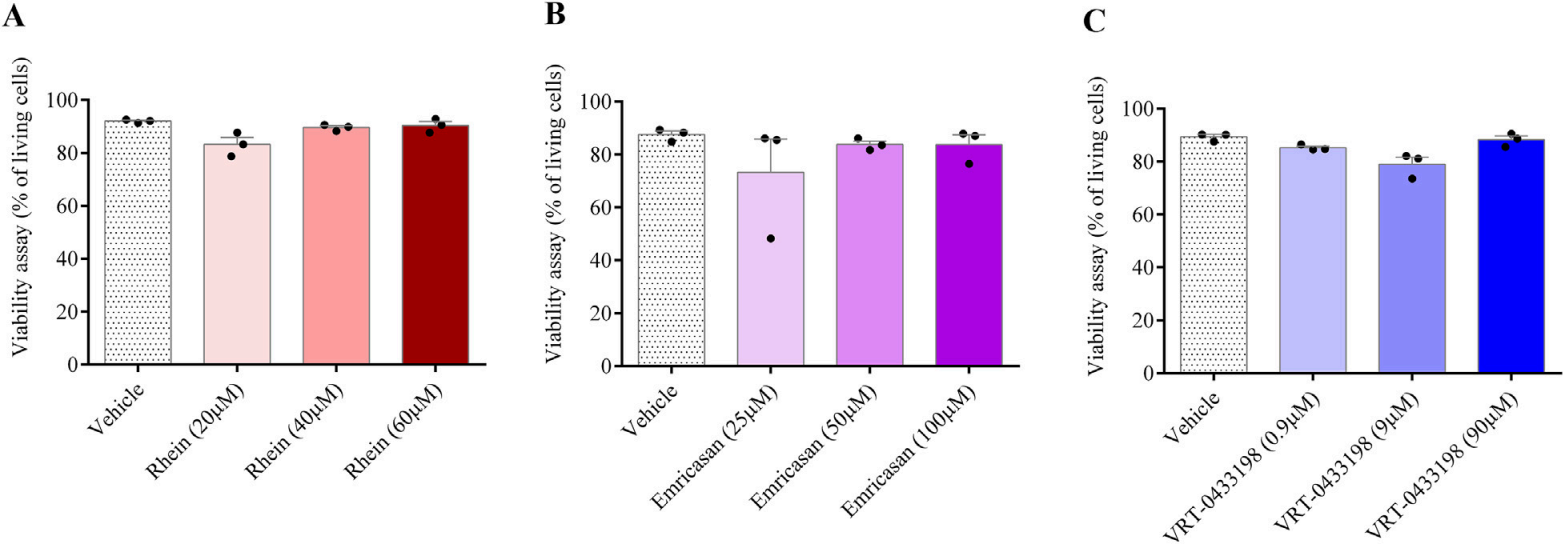
Non-toxic effects were verified in a cell viability assay. Human PBMCs were treated with different doses following 24-h treatment with rhein **(A)**, VRT043198 **(B)**, and emricasan **(C)**.

**FIGURE 2:**
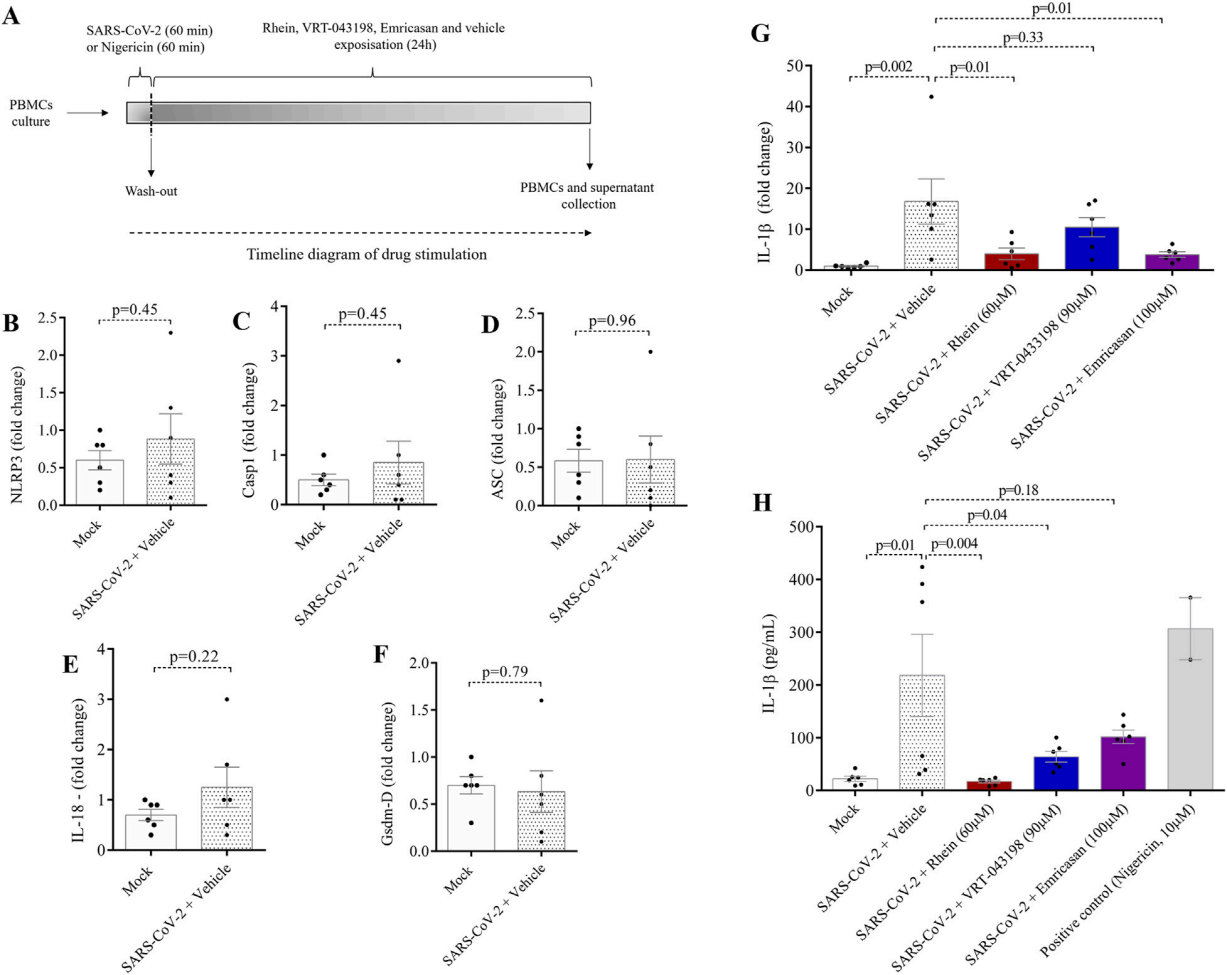
Rhein treatment reduces IL-1β gene expression and protein secretion in human PBMCs. Human PBMCs obtained from healthy volunteers were infected *in vitro* for 1 h under continuous agitation and stimulated with rhein, VRT-043198, emricansan or vehicle (dimethyl sulfoxide, DMSO) for 24 h. The supernatant and protein extract were obtained at the end of the protocol to analyze for inflammatory markers. **(A)** timeline diagram of infection and drug stimulation. Relative mRNA expression of **(B)** NLRP3, **(C)** caspase-1, **(D)** ASC, **(E)** IL-18, **(F)** gasdermin-D, and **(G)** IL-1β. **(H)** Measurement of IL-1β secretion by ELISA. The data represent the mean ± SEM of at least two independent experiments performed in triplicate.

We conducted *in vitro* experiments using rhein alongside two other caspase inhibitors: (i) VRT-043198, a specific caspase-1 inhibitor, and (ii) emricasan, a pan-caspase inhibitor. No cytotoxic effects of the inhibitors on human PBMCs were observed when conducting viability assays at three distinct doses over a 24-h exposure period (Figures 1A–C). Thus, we treated human PBMCs with rhein, emricasan, and VRT-043198 after SARS-CoV-2 infection, as shown in Figure 2A, and evaluated regulation of gene expression related to NLRP3 inflammasome proteins (Figures 2B–D). We also examined the gene expression of secondary pro-inflammatory markers, such as GSDM-D, IL-18, and IL1-β after SARS-CoV-2 infection (Figures 2E,F).

No significant changes in the gene expression of NLRP3 inflammasome proteins were observed following SARS-CoV-2 infection, as compared to the mock group in this experimental protocol (Figures 2B–D). Similarly, no changes were observed in the case of secondary markers like GSDM-D and IL-18 when comparing the SARS-CoV-2 group with the mock group (Figures 2E,F). However, a clear upregulation in IL-1β gene expression was evident in the SARS-CoV-2 group, consistent with findings in other studies (Figure 2G). In this scenario, both rhein and emricasan treatments led to a reduction in IL-1β transcript levels, whereas VRT-043198 did not demonstrate any significant effects on the assessed genes (Figure 2G). These findings indicate that rhein may have an anti-inflammatory effect by suppressing the mRNA levels of the key pro-inflammatory IL-1β.

To assess the effectiveness of rhein in mitigating pro-inflammatory signals, we conducted experiments to measure its capacity to decrease IL-1β secretion alongside VRT-043198 and emricasan (Figure 2H). In human PBMCs infected with SARS-CoV-2, only rhein significantly reduced IL-1β secretion, as determined using ELISA. Together, these results suggest that rhein decreases IL-1β production and secretion. In contrast, emricasan does not significantly reduce secretion IL-1β secretion and VRT-043198 appeared to act exclusively post-transcriptionally, leading to a reduction solely in IL-1β secretion (Figure 2H).

**FIGURE 3:**
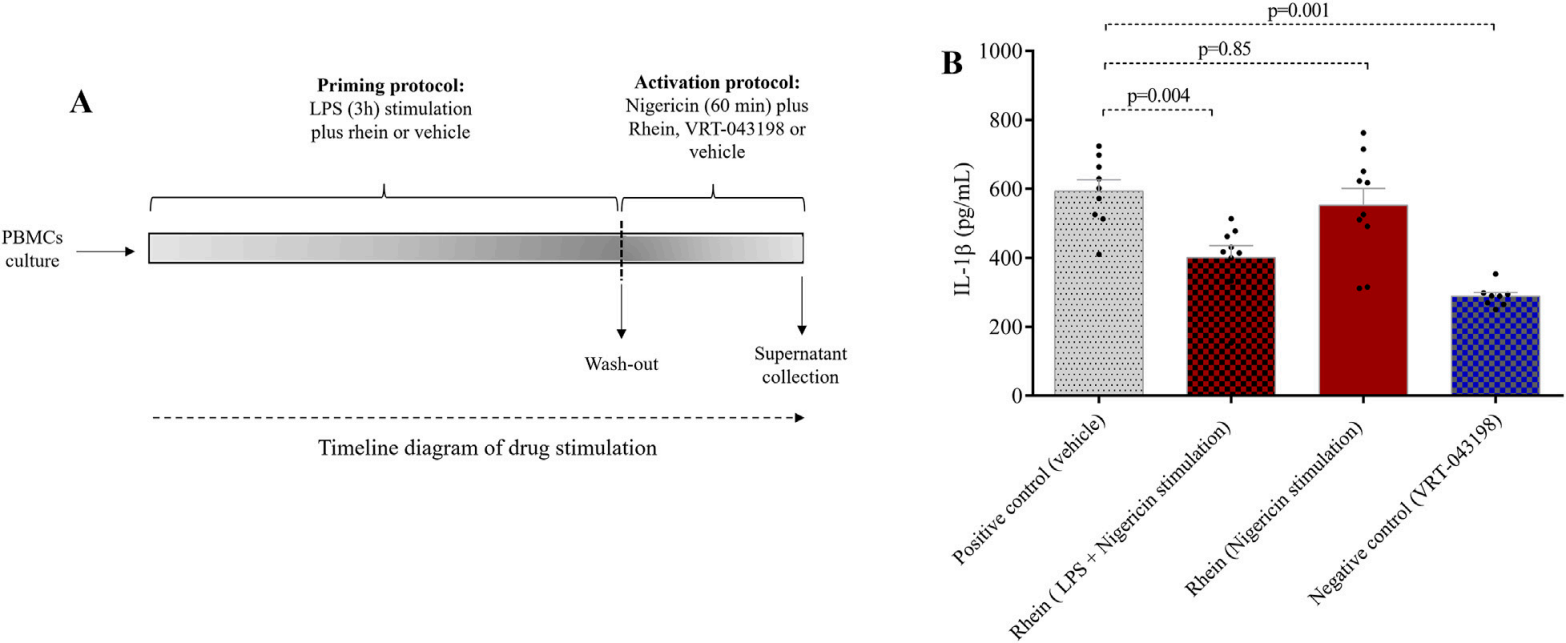
Rhein treatment under protocol of priming (or synthesis) stimulation and activation of NLRP3 inflammasome reduces IL-1β secretion in human PBMCs. Human PBMCs obtained from healthy volunteers were subjected to the NLRP3 inflammasome transcriptional and post transcription protocol also named priming and activation protocol and treated with rhein at two distinct time points. **(A)** timeline diagram of drug stimulation. **(B)** evaluation of IL-1β secretion under rhein treatment starts with lipopolysaccharide for 3 h of exposure plus nigericin for 1 h, or (ii) only with nigericin for 1 h. The positive control was the vehicle (dimethyl sulfoxide, DMSO) and the negative control was VRT-043198 (0.5 µM). The data represent the mean ± SEM of at least two independent experiments performed in triplicate.

**FIGURE 4:**
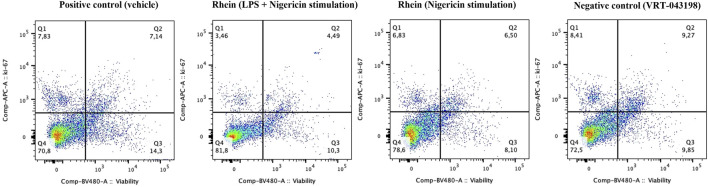
Non-toxic and non-proliferation inducer effects were verified during the priming and activation protocol. Human PBMCs were intracellularly labeled and analyzed using flow cytometry to assess toxicity and proliferation capacity in the presence of rhein (60 µM) and the positive control VRT-043198 (0.5 µM).

To elucidate the impact of rhein-mediated downregulation on IL-1β and explore rhein’s potential influence on NLRP3 inflammasome activation inhibition, we conducted experiments using human PBMCs and specific activators targeting transcriptional and post-transcriptional steps (Figure 3A). Rhein administration during the first stimulus significantly reduced IL-1β secretion compared to the control group, which encompassed the combined effects of both stimuli (Figure 3B). However, this inhibitory effect was absent when rhein was administered during the second stimulus (nigericin’s activation) (Figure 3B). In contrast, VRT-043198 administration during the second stimulus effectively inhibited IL-1β secretion (Figure 3B). These results indicate that rhein possibly interferes with NLRP3 inflammasome assembly and IL-1β transcription regulation but does not impede its NLRP3 inflammasome activity post-activation. Correspondingly, in an *in vitro* caspase-1 activity assay, we observed a modest direct reduction with rhein compared to its specific inhibitor VRT-043198 (quadruplicate tests: −18% ± 3% vs. −99 ± 1, respectively; *p* = 0.018). After 24 h, none of the groups exhibited cytotoxic effects or induction of proliferation that could have impacted the tests. This was confirmed through intracellular labelling and analyzed using flow cytometry (Figure 4).

### 3.2 Evaluation of inhibitory effects on SARS-CoV-2 replication in human PBMCs

**FIGURE 5:**
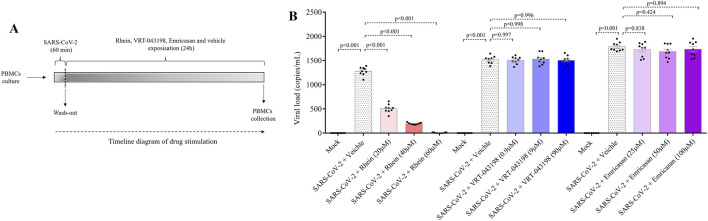
Rhein treatment reduces the viral load of SARS-CoV-2 in infected human PBMCs *in vitro*. Human PBMCs obtained from healthy volunteers were infected *in vitro* for 1 h under continuous agitation and stimulated with rhein, VRT-043198, Emricasan, or vehicle (dimethyl sulfoxide, DMSO) for 24 h. **(A)** Timeline diagram of drug stimulation. **(B)** Evaluation of viral load with inhibitors at different dose concentrations plus Mock and vehicle groups. The data represent mean ± SEM of at least two independent experiments performed in triplicate.

**FIGURE 6:**
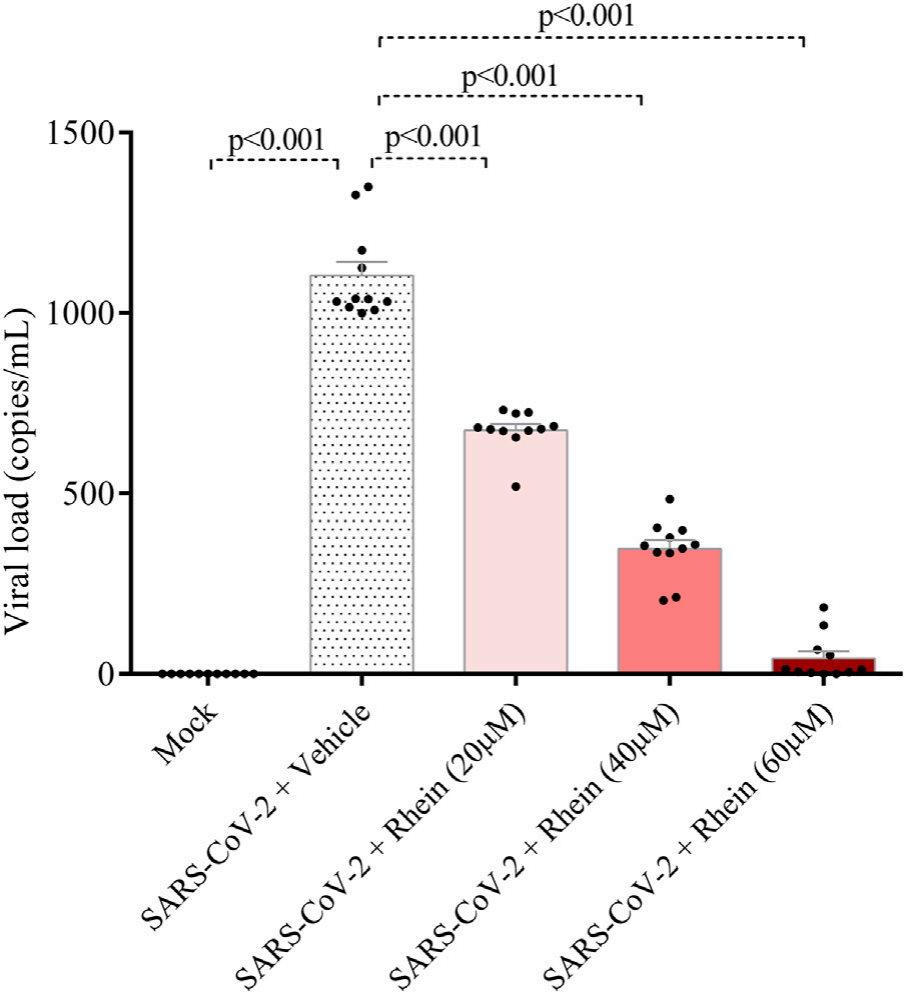
Rhein treatment reduces the viral load in human PBMCs prior to *in vitro* infection with SARS-CoV-2. Human PBMCs obtained from healthy volunteers were treated with rhein at different doses before being infected *in vitro* for 1 h under continuous agitation and stimulation.

We conducted experiments to explore the impact of rhein (20, 40 and 60 µM), emricasan (25, 50 and 100 µM), and VRT-0433198 (0.9, 9 and 90 µM) on viral replication in human PBMCs infected with SARS-CoV-2, as shown in Figure 5A. Our results demonstrated that emricasan and VRT-0433198 did not exhibit any inhibitory effect on viral replication, suggesting that the effect of rhein is independent of inflammasome activation (Figure 5B). In contrast, rhein treatment exhibited a dose-dependent reduction in viral load, decreasing close to zero viral copies detected even when administered either before (Figure 6).

### 3.3 Evaluation of the antiviral mechanism exerted by rhein

**FIGURE 7:**
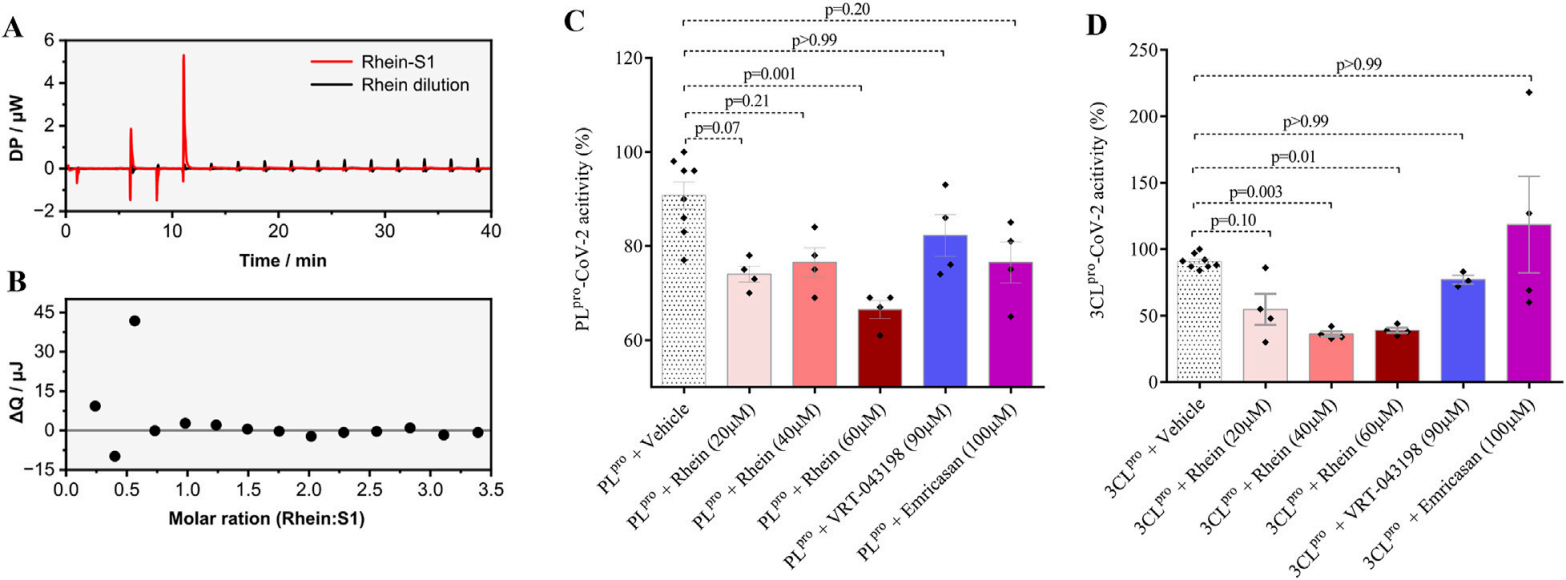
Rhein molecule has a low affinity for the S1 protein and significant pharmacological properties in reducing the activity of SARS-CoV-2 proteases 3CL^Pro^ and PL^pro^. The specific assay evaluated the interaction between rhein and the viral S1 protein revealed low binding energy between them. In parallel, rhein significantly reduced the activity of the proteases used by SARS-CoV-2 for genetic material replication. **(A)** baseline corrected raw isothermal titration calorimetry (ITC) data of rhein injection into S1 solution and rhein dilution in PBS. **(B)** binding isotherm of rhein-S1 after subtraction of the rhein heat of dilution. **(C)** Effect of rhein on the processing of non-structural virus proteins by 3CL^pro^-SARS-CoV-2, and **(D)** PL^pro^-SARS-CoV-2 with rhein, VRT-043198, emricansan or vehicle (dimethyl sulfoxide, DMSO). The data represent mean ± SEM of at least two independent experiments performed in triplicate.

As previously mentioned, there is a proposition that rhein may establish a covalent bond with the S1 domain of the Spike protein, thereby impeding viral envelope fusion (Ho et al., 2007). To investigate this hypothesis, we utilized isothermal titration calorimetry to verify the interaction between rhein and the S1 protein. Figure 7A displays the titration curves for rhein in the S1 solution and PBS (control), showing a few interaction peaks that could be associated with some external heat change. The peaks presented similar intensities. Figure 7B shows the energy variation after peak areas integration and dilution discount from the rhein-S1 curve. The observed small energy variations suggest negligible or weak interaction between rhein and S1 in the experimental conditions studied, with energy lower than 50 kJ/mol or lower/intermediate affinity.

We next evaluated the effect of rhein on the activity of two key enzymes used by SARS-CoV-2 for replication: the main protease (M^pro^), also known as 3-chymotrypsin-like protease SARS-CoV-2 3CL^pro^, and papain-like protease SARS-CoV-2 PL^pro^. These enzymes play a crucial role in processing new non-structural viral proteins (nsp), by cleaving certain regions of polyproteins (pp1a and pp1ab) synthesized from the transcription of the viral genome. Currently, 16 nsps are known, including the 3CL^pro^ enzymes (nsp5) and PL^pro^ (nsp3) (Zhu et al., 2020; Khan et al., 2021; Moustaqil et al., 2021). Consistently, rhein reduced the proteolytic activity of both 3CL^pro^-SARS-CoV-2 and PL^pro^-SARS-CoV-2 (Figures 7C,D).

### 3.4 Phosphoproteomics of human PBMC after SARS-CoV-2 infection with and without rhein

**FIGURE 8:**
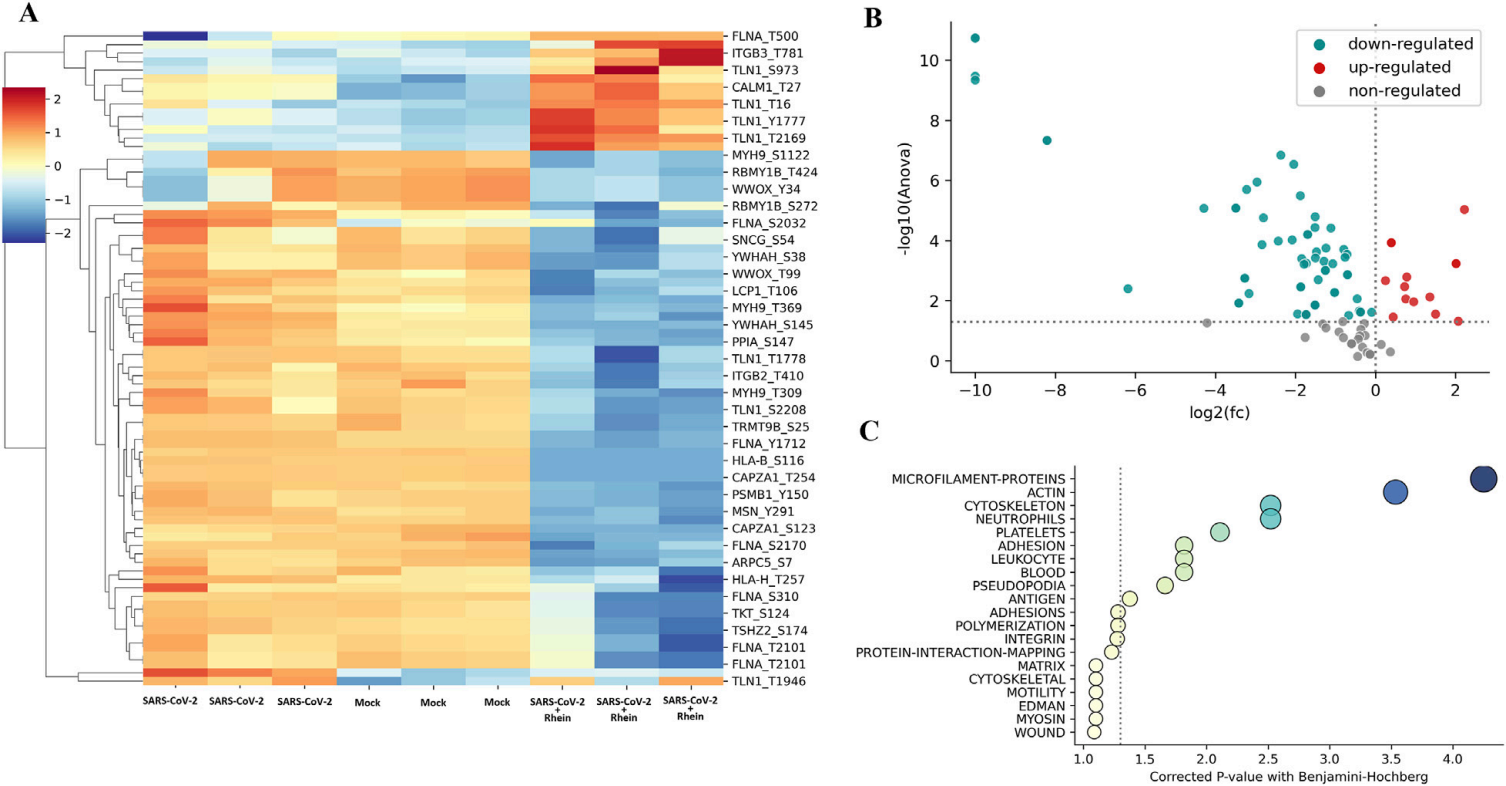
Rhein treatment can change phosphorylation patterns of extracellular matrix-related proteins in human PBMCs infected with SARS-CoV-2. A mass spectrometry-based phosphoproteomics assay revealed an increase in non-phosphorylated compared to phosphorylated proteins *in vitro*. **(A)** heatmap of phosphopeptides from human PBMCs infected with SARS-CoV-2, control (no treatment), and rhein treatment. **(B)** volcano plot of phosphopeptides that were found to be deregulated in control relative to rhein treatment. **(C)** the enrichment analysis of biological pathways with phosphopeptides and their respective phosphorylation sites (upstream or downstream).

Hierarchical cluster analysis, considering only the peak intensity of the phosphopeptides, showed a distinct separation between the groups (Figure 8A). When comparing the SARS-CoV-2 and mock groups, 29 phosphopeptides showed differential regulation. In the comparison between SARS-CoV-2 plus rhein and mock groups, 88 phosphopeptides were differentially regulated. In the comparison between SARS-CoV-2 and SARS-CoV-2 plus rhein, 70 phosphopeptides exhibited differential regulation. Interestingly, rhein treatment in SARS-CoV-2 infected cells was followed by downregulation of the majority of these 70 phosphopeptides (Figure 8B), suggesting that the drug may be blocking protein kinases or enhancing phosphatase activity (Supplementary Table S1).

We conducted Gene Ontology analysis on proteins with statistically deregulated phosphopeptides revealing enrichment in biological processes primarily associated with cytoskeleton and cell structure proteins (e.g., microfilament proteins, actin, adhesion, and pseudopodia) (Figure 8C). Filamin A (FLNA) protein exhibited the most affected phosphosite on this pathway in SARS-CoV-2-infected cells treated with rhein.

### 3.5 The clinical trial

Between April 6th and 14 December 2022, we conducted a prospective, randomized, double-blind, placebo-controlled pilot clinical trial involving 14 adult patients diagnosed with COVID-19, with 7 individuals assigned to each study arm (Table 2). As commented earlier, the study was halted with this sample size due to a substantial decrease in outpatients with mild to moderate COVID-19 symptoms who sought medical care. Eligible participants exhibited moderate COVID-19 symptoms, defined by their placement on the World Health Organization’s Clinical Progression Scale (WHO-CPS) as stages 4 or 5 (WHO, 2020). These patients were confirmed COVID-19 cases through reverse transcription polymerase chain reaction (RT-PCR) testing and were enrolled within 4 ± 2 days of symptom onset. Noteworthy, 71% of the participants had previously received systemic corticosteroids before randomization.

**TABLE 2:** Demographic data at admission. All comparisons were non-significant based on student T, Wilcoxon test or Chi-square test analyses. Systolic blood pressure (SBP); Diastolic blood pressure (SBP); Chronic obstructive pulmonary disease (COPD); Human immunodeficiency virus (HIV); Angiotensin II receptor blockers (ARB); Angiotensin converting enzyme inhibitor (ACEI).

	Variables	Placebo (n = 7)	Diacerein (n = 7)	*p*-value
Demographics	Age (years)	58 ± 16	64 ± 17	0.37
	Female (%)	4 (57%)	3 (43%)	0.90
	Ethnicity - White (%)	6 (86%)	4 (57%)	0.19
Physical examination	SBP (mmHg)	120 ± 12	131 ± 20	0.24
	DBP (mmHg)	70 ± 6	71 ± 14	0.84
	Heart rate (BPM)	82 ± 9	81 ± 20	0.92
	Oxygen saturation (%)	96 ± 2	97 ± 1	0.81
	Respiratory rate (breaths/min)	21 ± 1	21 ± 1	0.61
	Temperature (^°^C)	36.7 ± 0.39	36.5 ± 0.27	0.26
Symptoms	Fever	3 (43%)	2 (29%)	1.00
	Fatigue	3 (43%)	2 (29%)	1.00
	Throat pain	1 (14%)	3 (43%)	0.35
	Cough	7 (100%)	7 (100%)	1.00
	Anorexia	2 (29%)	0 (0%)	0.15
	Myalgia	1 (14%)	1 (14%)	0.58
	Dyspnea	2 (29%)	2 (29%)	0.57
	Sputum	1 (14%)	1 (14%)	1.00
	Diarrhea	0 (0%)	1 (14%)	0.37
Comorbidities	Arterial Hypertension	4 (58%)	6 (86%)	0.78
	Diabetes Mellitus	3 (43%)	4 (57%)	1.00
	Chronic kidney disease	2 (29%)	4 (57%)	0.27
	Heart Failure	3 (43%)	4 (57%)	0.29
	Prior acute coronary syndrome	2 (29%)	4 (57%)	0.59
	COPD	1 (14%)	0 (0%)	1.00
	Current Smoker	1 (14%)	0 (0%)	1.00
	Former Smoker	0 (0%)	1 (14%)	1.00
	Obesity	0 (0%)	2 (29%)	0.46
	HIV	0 (0%)	0 (0%)	1.00
	Prior tuberculosis	0 (0%)	0 (0%)	1.00
Medications in use	Steroids	5 (71.4%)	5 (71.4%)	1.00
	Statins	4 (57%)	6 (86%)	0.82
	ARB or ACEI	4 (57%)	4 (57%)	1.00
	Beta-blockers	2 (29%)	3 (43%)	0.56
	Metformin	2 (28.6%)	3 (43%)	1.00
	Insulin	2 (29%)	3 (43%)	0.56
Biochemical analysis	RT-PCR for COVID-19	7 (100%)	7 (100%)	1.00
	C-reactive protein (mg/dL)	2.2 ± 1.9	3.2 ± 3.0	0.32
	Dimer D (ng/dL)	628 ± 518	1,049 ± 600	0.35
	Troponin I (ng/mL)	15 ± 11	24 ± 19	0.32
	Time from symptoms onset to admission (days)	4.1 ± 1.9	3.9 ± 1.3	0.75
	Time from admission to discharge (days)	7.1 ± 4.7	7.8 ± 4.9	0.81

Baseline characteristics were equivalent between the two study groups, as detailed in Table 2. The median age of our participants was 62 ± 16 years, and 50% were women. All participants in the study had received COVID-19 vaccinations. With one having received one dose, four having received two doses, and nine having received three doses. The average number of doses administered was 2.6 ± 0.5 for the diacerein group and 2.6 ± 0.8 for the placebo group (*p* > 0.05). No disparities were detected in terms of clinical manifestations, vital signs, comorbidities, or medication usage prior to COVID-19. None of the patients had innate or acquired immunodeficiency. Furthermore, plasma high sensitivity C-reactive protein (hs-CRP), D-dimer, and Troponin I level upon admission were found to be comparable between the two study arms (Table 2). The durations from the onset of symptoms to admission were comparable in both study arms (Table 2). None of the patients experienced respiratory failure, thromboembolism, or in-hospital deaths.

The duration from admission to discharge was comparable between the placebo and diacerein groups (7.1 ± 4.7 vs 7.8 ± 4.9 days; *p* = 0.81). Throughout this period, we monitored changes in markers of inflammasome activation, including plasma levels of NLRP3, caspase-1, GSDM-D, and IL-1β. As mentioned earlier, samples were collected at the time of admission, the second, fifth, and 10th day of the therapy. At admission, no significant difference was detected in the levels of these biomarkers between arms (Table 3). According to the AUC analyses, patients treated with diacerein exhibited lower levels of NLRP3, caspase-1, and GSDM-D during hospitalization compared to those who received the placebo (Table 3). Unexpectedly, most patients maintained very low plasma concentrations of IL-1β throughout the study, consistently remaining below the detection limit of 3.9 pg/mL. This finding hinders our ability to confirm the effect of diacerein on IL-1β secretion in the trial.

**TABLE 3:** Diacerein treatment reduced plasma inflammasome markers during a randomized clinical trial. The comparisons were performed using the area under the curve (AUC) for values at D0, D2, D5, and D10, adjusted for baseline values to account for the mean effect. The analyses were conducted using rank covariance analysis (RANKOVA). The symbol^*^ indicates *p* < 0.001 diacerein compared to control.

	Variables	Placebo (n = 7)	Diacerein (n = 7)	*p*-value
*Caspase-1*	Admission	193 (329)	208 (261)	
	Day 2	278 (274)	205 (386)	
	Day 5	242 (392)	140 (124)	
	Day 10	235 (189)	95 (178)	
	AUC	825 (960)	424 (888)	0.001^*^
*Gasdermin-D*	Admission	12.6 (18.8)	10.1 (5.8)	
	Day 2	13.8 (33.9)	8.7 (8.4)	
	Day 5	12.0 (17.9)	7.9 (9.1)	
	Day 10	12.3 (12.1)	4.5 (7.9)	
	AUC	37.7 (68.3)	22.7 (22.9)	0.001^*^
*NLRP3*	Admission	15.0 (10.1)	16.6 (14.5)	
	Day 2	16.7 (35.3)	11.4 (9.5)	
	Day 5	18.8 (9.3)	12.4 (18.6)	
	Day 10	16.8 (12.6)	11.9 (9.2)	
	AUC	79.6 (79.1)	62.2 (70.6)	0.0001^*^

## 4 Discussion

This study aimed to assess the biological plausibility of diacerein/rhein as a hypothetical candidate for COVID-19 therapy. Drawing from preliminary studies, two hypotheses were considered: (i) a reduction in inflammasome activation and (ii) a decrease in viral replication. The study results, derived from a combination of *in vitro* experiments and a pilot clinical trial involving patients in the acute phase of COVID-19 confirmed both hypotheses.

As commented above, the NLRP3 inflammasome activation is vital in the innate immune response to pathogenic infections, promoting the release of pro-inflammatory interleukins to facilitate the recruitment of immune cells for containing the infection’s spread (Zhao and Zhao, 2020; Pandey and Zhou, 2022). RNA viruses like SARS-CoV-2, along with their viral N protein, have been shown to trigger the NLRP3 inflammasome in host cells (da Costa et al., 2019; Pan et al., 2021). This pro-inflammatory response, however, may become dysregulated, potentially exacerbating the immune response, and triggering self-destructive reactions. Therefore, modulating this signaling pathway has the potential to alleviate hyperinflammation and COVID-19 symptoms (Zeng et al., 2022). Our *in vitro* experiments indicate that rhein, the active metabolite of diacerein, mitigates the SARS-CoV-2-induced increase in the transcription rate and secretion of IL-1β, which is a potent pro-inflammatory cytokine and a product of NLRP3 inflammasome activation. This finding aligns with prior studies showing rhein’s inhibition of nuclear factor of kappa light polypeptide gene enhancer in B-cells inhibitor, alpha (IκBα) phosphorylation, thereby blocking nuclear factor kappa-stimulated gene transcription through post-translational modifications (PTMs) (Torina et al., 2015; Liu et al., 2021). Nonetheless, our *in vitro* protocol has limitations, as it was not possible to determine the specific transcriptional regulation pathway for all proteins involved in the pyroptosis pathway, through which rhein exerted its inhibitory effect.

In contrast to our earlier clinical trial conducted at the onset of the pandemic, which involved unvaccinated COVID-19 patients, the present study observed predominantly very low levels of IL-1β in most participants (Lukhna et al., 2022). Nonetheless, a significant decrease in plasma levels of key NLRP3 inflammasome markers, specifically NLRP3, caspase-1, and GSDM-D, was observed in COVID-19 patients who received a 10-day diacerein treatment compared to those who received placebo. The presence of these proteins in plasma indicates increased activity of the pyroptosis pathway. The protein complex formed by NLRP3, ASC, and caspase-1 is responsible for the cleavage of GSDM-D in the cytoplasm, initiating an oligomerization process and the formation of pores in the cell membrane. In this context, GSDM-D is considered the executor of pyroptosis, leading to lytic cell death. This outcome results in an exacerbation of inflammation and a worsening of the disease condition. Considering the mild inhibitory effect of rhein, when directly targeting caspase-1 *in vitro*, this outcome indicates a mechanism of action occurring before inflammasome activation, such as priming, and possibly its assembly. There is a clear association between the acute inflammatory response and the attenuation of late post-COVID manifestations. Therefore, both vaccination and therapies that mitigate the acute inflammatory response, as demonstrated in this study, should reduce the incidence of long-term COVID. This possibility warrants further investigation in future studies (Nalbandian et al., 2021).

Anthraquinones with a rhein-like structure have demonstrated the ability to diminish viral replication (de Oliveira et al., 2020; Li et al., 2007; Wang et al., 2018). A prior *in vitro* study using the influenza A virus model indeed showcased the effectiveness of rhein in diminishing viral replication (Wang et al., 2018). The mechanism hypothesized was that rhein would form a covalent bond with the virus Spike 1 (S1) protein, thereby preventing its binding to angiotensin-converting enzyme 2 (ACE2) in targeted cells (Ho et al., 2007). As a result, the viral envelope would not fuse with the cell membrane. We confirmed the rhein’s dose-dependent effect to reduce SARS-CoV-2 replication in human PBMCs infected *in vitro*. However, in contrast to the initial hypothesis (Ho et al., 2007), we found no binding between rhein and SARS-CoV-2’s S1 protein, as indicated by a small enthalpy interaction. Consistently, molecular docking investigations revealed low binding energy (ΔG -8.73 kcal/mol) between rhein and the fragmented spike protein (Basu et al., 2020). Notably, rhein’s inhibitory effect remains consistent whether administered to human PBMCs before or after SARS-CoV-2 infection. These findings lead us to hypothesize that its mechanism of action might involve the intracellular machinery rather than blocking the viral S1 protein’s binding with the cell membrane.

The “hijacking” of host cell machinery involves the actin and microtubule cytoskeleton and is a critical step in viral infection for proliferation and completing its life cycle (Aminpour et al., 2022). SARS-CoV-2 fusion with the cell membrane and movements to and from the nucleus are essentially dependent on microtubule actions (Aminpour et al., 2022). Additionally, SARS-CoV-2 induces infected human cells to produce virus-laden filopodia, extending towards neighboring cells for invasion and viral spread (Wen et al., 2020; Ng et al., 2004). The movement of immune cells towards an antigen-containing cell, releasing cytokines, depends on microtubule activity (Aminpour et al., 2022). Consequently, drugs such as Paxlovid block the cytoskeleton and reduce viral load in COVID-19 patients (Hammond et al., 2022). Similarly, the drug jasplakinolide plays a role in polymerizing and stabilizing actin filaments, thereby retaining the virus within the actin cortex and preventing its insertion into host cells (Owczarek et al., 2018). Likely, the inhibitory effect of diacerein on viral replication can be at least partially attributed to its ability to inhibit the phosphorylation of proteins associated with the cytoskeleton network, as we demonstrated in the phosphoproteomic analysis. Despite rhein not preventing the upregulation of FLNA-T500, all other evaluated isoforms were downregulated, which, in turn, could influence multiple transmembrane proteins involved in the virus’s interaction with actin filaments (Malathi et al., 2014; Tirupula et al., 2015).

Targeting interactions with RNA-binding proteins has been equally effective in curbing viral replication (Zhou et al., 2022). This mechanism disrupts the processing of nsp, a crucial step in generating new virions. Specifically, it impacts 3CL^pro^-SARS-CoV-2 and PL^pro^-SARS-CoV-2 proteases, the primary enzymes responsible for regulating nsp processing and facilitating the functional activity of the replication-transcription complex. In our study, we observed that rhein effectively inhibited both proteases in an *in vitro* assay, indicating a likely contribution to its inhibitory effect on SARS-CoV-2 replication.

The study employed a translational design to compare *in vitro* findings with outcomes from a pilot-phase clinical trial. However, the study took place amid the widespread implementation of COVID-19 vaccines, creating two notable limitations. Firstly, the limited number of participants may compromise result accuracy, potentially hindering the detection of subtle effects. The absence of severely ill patients, besides challenging the detection of IL-1β changes, may yield results that are not easily extrapolated across the entire spectrum of disease severity. Future research, involving larger and more diverse samples, is imperative to address these limitations and offer a comprehensive understanding of the intervention’s effects.

Several confounding factors warrant attention. Brazil has reported cases of all major COVID-19 variants, including Zeta, Gamma, Delta, and Omicron, with an estimated death toll of approximately 700,000 individuals. During the recruitment phase of the clinical trial in 2022, the Omicron variant and its sub-lineages were most prevalent (Alcantara et al., 2022). Despite the absence of a specific treatment protocol for outpatients with COVID-19 symptoms, medications such as anticoagulants, azithromycin, budesonide, colchicine, corticosteroids, and azithromycin were frequently used (Falavigna et al., 2022). Additionally, the contentious use of medications like hydroxychloroquine/chloroquine, ivermectin, and nitazoxanide must be considered as potential confounding factors in this and other clinical studies.

## 5 Conclusion

In summary, the data gathered in this study indicates that diacerein possesses mechanisms of action capable of reducing viral replication and mitigating the inflammatory response related to SARS-CoV-2. These findings serve as aspects of biological plausibility that warrant exploration in clinical trials to assess their clinical utility.

## Data Availability

The original contributions presented in the study are publicly available. This data can be found here: repository of University of Campinas, https://doi.org/10.25824/redu/YEO4QS.

## References

[B1] Alcantara L. C. J. Nogueira E. Shuab G. Tosta S. Fristch H. Pimentel V. (2022). SARS-CoV-2 epidemic in Brazil: how the displacement of variants has driven distinct epidemic waves. Virus Res. 315, 198785. 10.1016/j.virusres.2022.198785 35461905 PMC9022374

[B2] Aminpour M. Hameroff S. Tuszynski J. A. (2022). How COVID-19 hijacks the cytoskeleton: therapeutic implications. Life (Basel, Switzerland) 12 (6), 814. 10.3390/life12060814 35743845 PMC9225596

[B3] Basu A. Sarkar A. Maulik U. (2020). Molecular docking study of potential phytochemicals and their effects on the complex of SARS-CoV2 spike protein and human ACE2. Sci. Rep. 10 (1), 17699. 10.1038/s41598-020-74715-4 33077836 PMC7573581

[B4] Codo A. C. Davanzo G. G. Monteiro L. B. de Souza G. F. Muraro S. P. Virgilio-da-Silva J. V. (2020). Elevated glucose levels favor SARS-CoV-2 infection and monocyte response through a HIF-1α/Glycolysis-Dependent Axis. Cell Metab. 32, 498–499. 10.1016/j.cmet.2020.07.015 32877692 PMC7462530

[B5] da Costa L. S. Outlioua A. Anginot A. Akarid K. Arnoult D. (2019). RNA viruses promote activation of the NLRP3 inflammasome through cytopathogenic effect-induced potassium efflux. Cell Death Dis. 10 (5), 346. 10.1038/s41419-019-1579-0 31024004 PMC6483999

[B6] de Oliveira P. G. Termini L. Durigon E. L. Lepique A. P. Sposito A. C. Boccardo E. (2020). Diacerein: a potential multi-target therapeutic drug for COVID-19. Med. hypotheses 144, 109920. 10.1016/j.mehy.2020.109920 32534337 PMC7263256

[B7] Distler U. Kuharev J. Navarro P. Tenzer S. (2016). Label-free quantification in ion mobility-enhanced data-independent acquisition proteomics. Nat. Protoc. 11 (4), 795–812. 10.1038/nprot.2016.042 27010757

[B8] Dominguez I. Cruz-Gamero J. M. Corasolla V. Dacher N. Rangasamy S. Urbani A. (2021). Okur-Chung neurodevelopmental syndrome-linked CK2α variants have reduced kinase activity. Hum. Genet. 140 (7), 1077–1096. 10.1007/s00439-021-02280-5 33944995

[B9] Falavigna M. Belli K. C. Barbosa A. N. Zavascki A. P. Nastri A. Santana C. M. (2022). Brazilian guidelines for the treatment of outpatients with suspected or confirmed COVID-19. A joint guideline of the Brazilian association of emergency medicine (abramede), Brazilian medical association (amb), Brazilian society of angiology and vascular surgery (sbacv), Brazilian society of geriatrics and gerontology (sbgg), Brazilian society of infectious diseases (SBI), Brazilian society of family and community medicine (sbfmc), and Brazilian thoracic society (sbpt). Braz J. Infect. Dis. 26 (2), 102347. 10.1016/j.bjid.2022.102347 35341739 PMC8926872

[B10] Hammond J. Leister-Tebbe H. Gardner A. Abreu P. Bao W. Wisemandle W. (2022). Oral nirmatrelvir for high-risk, nonhospitalized adults with covid-19. New England J. Med. 386 (15), 1397–1408. 10.1056/NEJMoa2118542 35172054 PMC8908851

[B11] Henamayee S. Banik K. Sailo B. L. Shabnam B. Harsha C. Srilakshmi S. (2020). Therapeutic emergence of rhein as a potential anticancer drug: a review of its molecular targets and anticancer properties. Molecules 25 (10), 2278. 10.3390/molecules25102278 32408623 PMC7288145

[B12] Ho T. Y. Wu S. L. Chen J. C. Li C. C. Hsiang C. Y. (2007). Emodin blocks the SARS coronavirus spike protein and angiotensin-converting enzyme 2 interaction. Antivir. Res. 74 (2), 92–101. 10.1016/j.antiviral.2006.04.014 16730806 PMC7114332

[B13] Kazmierski J. Friedmann K. Postmus D. Emanuel J. Fischer C. Jansen J. (2022). Nonproductive exposure of PBMCs to SARS-CoV-2 induces cell-intrinsic innate immune responses. Mol. Syst. Biol. 18 (8), e10961. 10.15252/msb.202210961 35975552 PMC9382356

[B14] Khan M. T. Irfan M. Ahsan H. Ahmed A. Kaushik A. C. Khan A. S. (2021). Structures of SARS-CoV-2 RNA-binding proteins and therapeutic targets. Intervirology 64 (2), 55–68. 10.1159/000513686 33454715 PMC7900486

[B15] Li Z. Li L. J. Sun Y. Li J. (2007). Identification of natural compounds with anti-hepatitis B virus activity from Rheum palmatum L. ethanol extract. Chemotherapy 53 (5), 320–326. 10.1159/000107690 17785969

[B16] Liu M. Wang L. Wu X. Gao K. Wang F. Cui J. (2021). Rhein protects 5/6 nephrectomized rat against renal injury by reducing inflammation via NF-κB signaling. Int. Urol. Nephrol. 53 (7), 1473–1482. 10.1007/s11255-020-02739-w 33763781

[B17] Lukhna K. do Carmo H. R. P. Castillo A. R. Davidson S. M. Geffen H. Giesz S. (2022). Effect of remote ischaemic conditioning on the inflammatory cytokine cascade of COVID-19 (RIC in COVID-19): a randomized controlled trial. Cardiovasc. Drugs Ther. 38, 433–445. 10.1007/s10557-022-07411-2 36445625 PMC9707178

[B18] Malathi K. Siddiqui M. A. Dayal S. Naji M. Ezelle H. J. Zeng C. (2014). RNase L interacts with Filamin A to regulate actin dynamics and barrier function for viral entry. mBio 5 (6), e02012. 10.1128/mBio.02012-14 25352621 PMC4217177

[B19] Manunta M. D. I. Lamorte G. Ferrari F. Trombetta E. Tirone M. Bianco C. (2021). Impact of SARS-CoV-2 infection on the recovery of peripheral blood mononuclear cells by density gradient. Sci. Rep. 11 (1), 4904. 10.1038/s41598-021-83950-2 33649400 PMC7921094

[B20] Moustaqil M. Ollivier E. Chiu H. P. Van Tol S. Rudolffi-Soto P. Stevens C. (2021). SARS-CoV-2 proteases PLpro and 3CLpro cleave IRF3 and critical modulators of inflammatory pathways (NLRP12 and TAB1): implications for disease presentation across species. Emerg. Microbes Infect. 10 (1), 178–195. 10.1080/22221751.2020.1870414 33372854 PMC7850364

[B21] Nalbandian A. Sehgal K. Gupta A. Madhavan M. V. McGroder C. Stevens J. S. (2021). Post-acute COVID-19 syndrome. Nat. Med. 27 (4), 601–615. 10.1038/s41591-021-01283-z 33753937 PMC8893149

[B22] Ng M. L. Lee J. W. Leong M. L. Ling A. E. Tan H. C. Ooi E. E. (2004). Topographic changes in SARS coronavirus-infected cells at late stages of infection. Emerg. Infect. Dis. 10 (11), 1907–1914. 10.3201/eid1011.040195 15550199 PMC3328989

[B23] Owczarek K. Szczepanski A. Milewska A. Baster Z. Rajfur Z. Sarna M. (2018). Early events during human coronavirus OC43 entry to the cell. Sci. Rep. 8 (1), 7124. 10.1038/s41598-018-25640-0 29740099 PMC5940804

[B24] Pan P. Shen M. Yu Z. Ge W. Chen K. Tian M. (2021). SARS-CoV-2 N protein promotes NLRP3 inflammasome activation to induce hyperinflammation. Nat. Commun. 12 (1), 4664. 10.1038/s41467-021-25015-6 34341353 PMC8329225

[B25] Pandey K. P. Zhou Y. (2022). Influenza A virus infection activates NLRP3 inflammasome through trans-golgi network dispersion. Viruses. 14 (1), 88. 10.3390/v14010088 35062292 PMC8778788

[B26] Rodrigues T. S. de Sa K. S. G. Ishimoto A. Y. Becerra A. Oliveira S. Almeida L. (2021). Inflammasomes are activated in response to SARS-CoV-2 infection and are associated with COVID-19 severity in patients. J. Exp. Med. 218 (3), e20201707. 10.1084/jem.20201707 33231615 PMC7684031

[B27] Tirupula K. C. Ithychanda S. S. Mohan M. L. Naga Prasad S. V. Qin J. Karnik S. S. (2015). G protein-coupled receptors directly bind filamin A with high affinity and promote filamin phosphorylation. Biochemistry 54 (44), 6673–6683. 10.1021/acs.biochem.5b00975 26460884 PMC4642222

[B28] Torina A. G. Reichert K. Lima F. de Souza Vilarinho K. A. de Oliveira P. P. do Carmo H. R. (2015). Diacerein improves left ventricular remodeling and cardiac function by reducing the inflammatory response after myocardial infarction. PloS one 10 (3), e0121842. 10.1371/journal.pone.0121842 25816098 PMC4376692

[B29] Vora S. M. Lieberman J. Wu H. (2021). Inflammasome activation at the crux of severe COVID-19. Nat. Rev. Immunol. 21 (11), 694–703. 10.1038/s41577-021-00588-x 34373622 PMC8351223

[B30] Wang Q. W. Su Y. Sheng J. T. Gu L. M. Zhao Y. Chen X. X. (2018). Anti-influenza A virus activity of rhein through regulating oxidative stress, TLR4, Akt, MAPK, and NF-κB signal pathways. PloS one 13 (1), e0191793. 10.1371/journal.pone.0191793 29385192 PMC5791991

[B31] Wen Z. Zhang Y. Lin Z. Shi K. Jiu Y. (2020). Cytoskeleton-a crucial key in host cell for coronavirus infection. J. Mol. Cell Biol. 12 (12), 968–979. 10.1093/jmcb/mjaa042 32717049 PMC7454755

[B32] WHO (2020). A minimal common outcome measure set for COVID-19 clinical research. Lancet Infect. Dis. 20 (8), e192–e197. 10.1016/S1473-3099(20)30483-7 32539990 PMC7292605

[B33] Zeng J. Xie X. Feng X. L. Xu L. Han J. B. Yu D. (2022). Specific inhibition of the NLRP3 inflammasome suppresses immune overactivation and alleviates COVID-19 like pathology in mice. EBioMedicine 75, 103803. 10.1016/j.ebiom.2021.103803 34979342 PMC8719059

[B34] Zhao C. Zhao W. (2020). NLRP3 inflammasome-A key player in antiviral responses. Front. Immunol. 11, 211. 10.3389/fimmu.2020.00211 32133002 PMC7040071

[B35] Zhou Y. Gao C. Vong C. T. Tao H. Li H. Wang S. (2022). Rhein regulates redox-mediated activation of NLRP3 inflammasomes in intestinal inflammation through macrophage-activated crosstalk. Br. J. Pharmacol. 179 (9), 1978–1997. 10.1111/bph.15773 34882785

[B36] Zhu W. Xu M. Chen C. Z. Guo H. Shen M. Hu X. (2020). Identification of SARS-CoV-2 3CL protease inhibitors by a quantitative high-throughput screening. ACS Pharmacol. Transl. Sci. 3 (5), 1008–1016. 10.1021/acsptsci.0c00108 33062953 PMC7507806

